# Process evaluation of a cross-sectoral, coordinated follow-up care of stroke patients: a qualitative study

**DOI:** 10.1186/s42466-024-00360-1

**Published:** 2025-01-23

**Authors:** Theresa Schrage, Claudia Glissmann, Götz Thomalla, David Leander Rimmele, Holger Schmidt, Michael Rosenkranz, Stefan Boskamp, Martin Härter, Levente Kriston

**Affiliations:** 1 Department of Medical Psychology, University Medical Center Hamburg Eppendorf, Martinistraße 52, 20246 Hamburg, Germany; 2 Department of Neurology, University Medical Center Hamburg Eppendorf, Martinistraße 52, 20246 Hamburg, Germany; 3 Department of Neurology, Luzerner Kantonsspital, Lucerne, Switzerland; 4 Department of Neurology, Elbe Klinikum Stade, Bremervörder Str. 111, 21682 Stade, Germany; 5 Department of Neurology and Neurological Early Rehabilitation, Albertinen Krankenhaus, Süntelstraße 11a, 22457 Hamburg, Germany

**Keywords:** Stroke, Follow-up care, Rehabilitation, Implementation analysis, Qualitative content analysis, Process evaluation

## Abstract

**Background:**

Implementation of interventions to improve follow-up stroke care is complex due to the involvement of various stakeholders and challenges of health care coordination. The aim of this study was to evaluate the process of implementing a cross-sectoral, coordinated follow-up care for stroke patients (the StroCare intervention).

**Methods:**

As part of a multicenter interventional trial, this qualitative study was performed in a pre-post design with semi-structured interviews conducted with patients and health care employees. The multicomponent intervention was implemented in eight participating acute care and rehabilitation clinics. The interviews were analyzed using qualitative content analysis. Contents were coded using eight a priori defined categories (acceptability, adoption, appropriateness, feasibility, fidelity, sustainability, patient-centeredness, satisfaction with treatment, and pandemic-related effects) with the possibility of inductively developed categories.

**Results:**

Interviews with 21 patients and 34 interviews with 23 employees were conducted. In addition to the deductive categories, three inductive categories (psychosocial implications, interconnectedness, and potential for improvement) emerged. Acceptability, adoption, and appropriateness were assessed positively before the intervention. However, poor feasibility had a negative impact on adoption and appropriateness. In contrast, outcomes related to patient care (patient-centeredness and psychosocial implications) were independent from this effect.

**Conclusions:**

Similar to other implementation studies of stroke care interventions, implementation of eHealth Services in the StroCare project met barriers in usability and adaptability of new software. However, high adoption and appropriateness in regard to patient-centeredness, psychosocial implications, and an overall benefit for the patients supported continuation of the remaining intervention components.

*Trial registration* The trial is registered at ClinicalTrials.gov (NCT04159324), registration date 12/11/19.

**Supplementary Information:**

The online version contains supplementary material available at 10.1186/s42466-024-00360-1.

## Background

Stroke is a leading cause of disability and death in Germany [1]. In 2017, more than a million people experienced a stroke incident in the European Union [2]. A stroke can lead to a wide range of physical and psychological symptoms as physical disability, depressive symptoms, and impairments in daily life, which can result in a decreased health-related quality of life [3–7]. Up to date, patients’ needs after stroke are often not sufficiently met by health care systems [8, 9].

Follow-up care for stroke patients has several objectives, including the restoration and improvement of lost cognitive functions, prevention of a recurrent stroke (secondary prevention), reintegration into daily routine, and an improvement of health-related quality of life [8, 10, 11]. Follow-up care encompasses in-patient and out-patient rehabilitation. At present, follow-up care is often not continuous because patients often have to wait between two follow-up treatments when a direct aftercare would be more beneficial [8]. Additionally, multisectoral aftercare is hindered by several barriers [8]. First, in Germany health care capacities for neurologic inpatient rehabilitation are limited, and it takes time until cost coverage applications are processed by health insurance companies. This results in patients often having to wait longer than recommended after acute care to begin inpatient rehabilitation [12]. Second, the subsequent outpatient care after discharge is often delayed and not well coordinated. Patients often have to wait a long time for outpatient appointments with a neurologist, are unaware of possible and necessary therapies (for example physical, logopedic, or occupational therapy), and are in need of help to organize admission to these services.

Cross-sectional stroke aftercare is receiving continuously increasing research interest both in and outside Germany. Although some studies suggest positive effects of various interventions [13–15], others report no substantial improvement of clinical outcomes after implementing novel aftercare concepts [16–21]. At the same time, research synthesis and evidence transfer is challenged by substantial differences in health care systems across countries. For example, the strict separation of inpatient and the outpatient care sector is a major obstacle to integrated stroke care in Germany. Although the situation is somewhat more favorable in some European countries, considering the “entire chain of care” is a central aim of the Action Plan for Stroke in Europe [22].

To improve follow-up care of stroke patients in Germany, a team of diverse health care stakeholders developed and executed the comprehensive and cross-sectoral intervention StroCare [23]. Three acute care and five rehabilitation clinics, complemented by a case manager, participated to provide a coordinated and evidence-based care. The project aimed at implementing five major intervention components into routine care:

1. **Primary contact:** A study nurse was appointed as the primary contact for each patient, making contact per telephone every three to six months for two years and organizing outpatient follow-up appointments at the acute care clinics. The primary contact person also took part in the follow-up appointments.
2. **Outpatient care management**: Follow-up appointments with study nurses and neurologists at the acute care clinics were organized every six months for two years, including a neurological examination, duplex ultrasound of extracranial and intracranial brain supplying arteries, evaluation of cardiovascular risk factors, setting of treatment goals, standardized collection of patient-reported outcomes, and planning of further treatment.
3. **Case management:** Every patient was allocated to a case manager who assisted the patient for one year with various health concerns, from submission of health care applications for health care services to organizing outpatient therapies according to the patient’s treatment goals.
4. **Coverage of costs:** Patients participating in this project all were a member of one health insurance organization, which committed itself to cover the cost of inpatient rehabilitation.
5. **Electronic allocation of capacities:** An electronic rehabilitation portal was implemented to speed up and ensure tailored allocation to the five cooperating rehabilitation clinics. The portal should also ensure efficient information transfer (medical reports, images, diagnoses) between the acute care and the rehabilitation clinics, as well as the case management.

In the present study, we aimed to explore and evaluate the process of implementing the StroCare intervention components from the perspective of patients, health care professionals (HCPs), study coordinators, and IT staff. We intended to specifically understand their experiences with implementation outcomes such as acceptability and feasibility of the intervention. In addition, we were also interested in subjectively perceived effects of the intervention, for example on patient-centeredness and satisfaction with treatment. Additionally, we aimed to understand the change in the staff’s assessment towards the implementation outcomes and intervention effects from before to after implementation of StroCare.

## Methods

This study was part of the multicenter StroCare project, which aimed to develop, implement, and evaluate a complex cross-sectoral, coordinated, and evidence-based intervention to improve stroke care [23]. The aim of the present study was to explore and evaluate the implementation process and to assess subjectively experienced intervention effects that are closely interconnected with the process evaluation. Additionally, change in the staff’s assessment towards the implementation outcomes and intervention effects was evaluated by a pre-post comparison. The trial was registered at ClinicalTrials.gov (NCT04159324) and conducted in accordance with the Declaration of Helsinki. The local ethics committees (review boards of the physician chambers of Hamburg, Niedersachsen, and Schleswig–Holstein, Germany) approved the study design.

### Study design

The present study was conducted in all study centers of the StroCare project and had a qualitative design. Interviews with patients were conducted after implementation of the StroCare intervention, and interviews with staff before and after implementation. For evaluation of the implementation process and intervention effects, a qualitative content analysis of semi-structured interviews conducted with patients and hospital staff (study nurses, neurologists, study coordinators, IT staff) was performed [24]. All participants provided written informed consent.

### Recruitment of participants

Patients were enrolled during their initial stay in the stroke unit. As the StroCare study had a sequentially controlled design with a control and an intervention phase, patients were allocated to the control or intervention group depending on the time of their admission. Recruitment of the control group was planned for 12 months, followed by recruitment of the intervention group for the next 12-month period. Due to effects of the Corona pandemic, less stroke patients were admitted to the acute care clinics. Thus, the recruitment period was extended to 15 months for the control group and to 18 months for the intervention group. Enrollment of patients started in March 2020. Qualitative interviews were conducted with patients in the intervention group one year after enrollment. The employees (study nurses, neurologists, study coordinators and IT staff) were interviewed before and after the intervention was implemented. A sample size of 21 patients and 20 employees from different departments (acute clinic: 2 physicians, 2 study nurses, and 1 IT staff; rehabilitation clinic: 2 physicians, 1 study nurse or coordinator) was considered a sufficient sample size for this study [25].

The staff participating in the interviews were involved in the StroCare study and worked at the collaborating clinics. Patients had to meet the following criteria to participate in the study: treatment at one of the participating acute care clinics; diagnosis (ICD-10) of ischemic attack (I63), transient ischemic attack and related syndromes (G45), or intracerebral haemorrhage (I64); insurance with the BARMER health insurance agency; and sufficient mastery of German language. Exclusion criteria were premorbid score of modified Rankin Scale mRS ≥ 4, present diagnosis of artificial respiration (Z99.1); dementia (F00.x., F01.x. or G30.x) or aphasia (R47); substantially impaired communication capacity due to aphasia or dementia; and admission to a nursing home following the acute treatment. After admission to the stroke unit, study nurses screened potentially eligible patients using the electronic medical record, and in case of eligibility invited them to participate in the study.

### Measurements and analysis

Sociodemographic data were gathered by self-report. A semi-structured interview guide was developed according to Helferrich [26] [see Additional File 1]. Questions were based on the proposed indicators for implementation analysis by Proctor and colleagues [27]. In addition, questions related to patient-centeredness of the interventions, satisfaction with the treatment, and effects of the Corona-pandemic on the implementation were included.

The analysis was conducted using qualitative content analysis based on Mayring [24], using categories derived from literature (deductive) and from the interview transcripts (inductive). Deductive categories were acceptance, adoption, appropriateness, feasibility, fidelity, and sustainability. In addition, categories for patient-centeredness, satisfaction with treatment, and pandemic-related effects were used to explore intervention effects. Inductive categories that emerged from the text material concerned psychosocial implications, interconnectedness, and potential for improvement. More detailed information can be found in the supplements [Additional File 2]. The category system is presented in Table 1.

**Table 1:** Domains of the qualitative content analysis

Category	Higher-level domain	Definition
*Deductive categories*		
Acceptability	Implementation outcome	An implementation is accepted if those involved or affected are satisfied with the process and/or the result of the realized innovation. The acceptance of different intervention components can vary and change dynamically over time as a result of experience.
Adoption	Implementation outcome	Adoption is given if there is a certain motivation to implement the planned innovation, i.e., the stakeholders show the intention to initiate the planned innovation and feel commitment to it.
Appropriateness	Implementation outcome	Appropriateness describes how compatible the innovation is with the actual conditions of practice. An appropriate innovation is experienced as relevant, suitable, and useful. However, the actual implementation of an appropriate innovation can also be poorly accepted, which allows a distinction to acceptability.
Feasibility	Implementation outcome	Feasibility of an innovation shows whether it could be successfully applied. To do so, it is insufficient to see the innovation as useful; it also needs to be practicable. For example, sufficient resources and information must be provided for those involved in the implementation process.
Fidelity	Implementation outcome	Fidelity is achieved, if the innovation can be implemented as intended by the developers, and adherence to the protocol is given.
Sustainability	Implementation outcome	Sustainability is considered given, if the changes can be institutionalized into routine procedures, and if continuation, integration, and routinization are possible and beneficial.
Patient-centeredness	Intervention effect	Patient-centeredness can be operationalized through many individual aspects. These include communication skills on the part of the practitioner, involvement of a patient in decision-making, accessibility to care, as well as consideration of all possible treatment options.
Satisfaction with treatment	Intervention effect	Satisfaction with treatment reflects the general perception and evaluation of stroke treatment.
Pandemic-related effects	Intervention effect	Due to the prevailing COVID-19 pandemic and the associated organizational changes in hospital care, restrictions were imposed, so that treatment could have been affected. These changes are targeted in this category.
*Inductive categories*		
Psychosocial implications	Intervention effect	Psychosocial effects include psychological symptoms such as anxiety or depressive symptoms, but also include other psychological constructs like quality of life and self-efficacy.
Interconnectedness	Intervention effect	Interconnectedness is achieved through fast and direct communication of relevant data between all care providers involved. The inter-sectoral exchange of information requires an efficient communication within the network of caregivers.
Potential for improvement	Implementation outcome, intervention effect	Improvement opportunities arise from the experience gained during implementation. Certain aspects of the innovations may be more helpful and implementable or new ideas that contribute to improving stroke care in the future have been generated.

## Results

### Study sample

As intended, 21 patients were interviewed after implementation of the intervention (Table 2). The mean age of the patients was 74 years (standard deviation, SD, 10.1 years), with more than 50% female participants. Most of the participating patients (70%) reported secondary school as their highest level of education, and 30% reported a university entrance qualification.

**Table 2:** Sample description of the interviewed patients (*N* = 20)*

	Absolute frequency	Relative frequency
Age, mean (*SD*)	74.24 (10.10)	
*Gender*		
Female	12	0.60
Male	8	0.40
*School graduation*		
No high school degree	14	0.70
High school degree	6	0.30
*Professional degree*		
Apprenticeship	13	0.65
University diploma (Bachelor, Master, Diploma)	3	0.15
Other	4	0.20
Employment status		
(Self-) Employed	2	0.10
Retired	18	0.90
*Relationship status*		
Without permanent relationship	8	0.40
Married or in a permanent relationship	12	0.60
*Children*		
Yes	14	0.70
No	6	0.30
*Living situation*		
Alone	8	0.40
Living with partner	12	0.60

^*^Data are missing for one of the 21 interviewed patients

Due to rotation schedule of the staff, the sample of interviewees before the intervention was not identical to the sample of interviewees after the intervention. Nine employees were interviewed only before implementing the StroCare intervention, four only after implementation, and ten both before and after implementation, summing up to a total of 20 interviews before and 14 interviews after implementation. Less interviews were conducted after implementation because less employees were engaged with StroCare during the intervention phase of the StroCare project. In the sample of 24 employees participating in the interviews, the mean age was 45 years (SD 9.54 years; Table 3). The majority of the sample worked as study nurses (33%) or neurologists (38%), with a mean work experience of 19 years.

**Table 3:** Sample description of the interviewed employees (*N* = 23)

	Absolute frequency	Relative frequency
Age, mean (SD)	45.21 (9.54)	
*Gender*		
Female	13	0.54
Male	11	0.46
*Clinic*		
Acute care clinic	17	0.74
Rehabilitation clinic	6	0.26
Work experience in years, mean (SD)	18.92 (11.34)	
*Professional group*		
Study nurse	8	0.33
Neurologist	9	0.38
IT staff	3	0.13
Study coordinator	4	0.17

### Qualitative content analysis

#### Acceptability

Acceptance of the StroCare interventions was generally neutral to positive. The outpatient care management in particular achieved a high level of acceptance among patients. The sufficient flow of information and planning of resources within the treatment teams of each clinic, and the perceived positive effects on patient interaction and the treatment of patients were viewed as positive.

#### Adoption

There was a general commitment to the implementation of the StroCare intervention, which was often explained by the perceived usefulness of the intervention components for patient care.

#### Appropriateness

One of the most important categories among the implementation outcomes was appropriateness. The continuous support and guidance throughout the aftermath of the stroke incident, specifically referring to the outpatient follow-up care and the case management were deemed highly relevant for patient stroke care. On the other side, the interventions also were sometimes deemed unnecessary in cases of a mild stroke.

#### Feasibility

The time required for and the pacing of the appointments were considered manageable by patients. On the contrary, hospital staff assessed the feasibility of the intervention as limited. Two main barriers were the two software programs for patient allocation to the rehabilitation clinics and for data entry and the selective inclusion of patients into the study. The selective inclusion in the study was a barrier to developing routine procedures. Further, problems with the software programs included a delayed implementation, inconvenient registration process, counterintuitive interface, and technical difficulties. While legislative challenges could be solved and the available hardware were suitable, most software components of the intervention were not ready to use during the project.

#### Fidelity

For the most part, the intervention components were carried out according to protocol. Regarding the electronic patient allocation platform, staff members returned to communication as usual.

#### Sustainability

All StroCare intervention components were deemed sustainable with the exception of the patient allocation platform.

#### Patient-centeredness

The StroCare intervention was reported to have positively influenced several dimensions of patient-centeredness, such as the clinician-patient-relationship, seeing the patient as a unique person, integration of medical and non-medical care, access to care, patient involvement in care, and emotional support.

#### Satisfaction with treatment

The patients deemed their satisfaction with treatment positive. For members of the staff, the modified follow-up care was met with an overall satisfaction, especially concerning the improved neurological care after rehabilitation.

#### Psychosocial implications

Several psychosocial effects seem to be connected to the StroCare intervention. These effects were the reduction of fear and of the feeling of loneliness, an increase in the sense of security and of a competence to act, mood enhancement and a stronger feeling of control, as well as a reduction of stress and of feeling of vulnerability. Patients and staff members explained the effects by the pre-planned and set structure of the follow-up care. In addition, the medical examinations were reported to enhance patients’ feeling of control and to decrease perceived health-related stress. Having a primary contact person enabled the patients to feel secure in their treatment.

#### Interconnectedness

For the most part, connectedness of the acute care hospitals, the rehabilitation clinics, and the case manager could not be increased due to considerable difficulties in the implementation of the patient allocation platform, which should have been the key element of StroCare for this goal.

#### Pandemic-related effects

Regarding possible effects of the Corona pandemic, few changes in the care provided were noted, but none had a major impact on the implementation of the intervention.

#### Potential for improvement

Development opportunities were largely identified for IT-related topics of patient care. The patient allocation platform would be helpful as an application for tablets and including a direct link to the hospital information system. Additionally, it was stated that access for other HCPs responsible for outpatient follow-up care (physiotherapists and logotherapists), a chat feature with HCPs, and digital health applications should be integrated into the platform.

### Contrasting assessments before and after implementation

The views and expectations of the employees before implementation of the intervention and one year after implementation differed. The categories adoption, appropriateness, feasibility, fidelity, and psychosocial implications indicated a change in attitudes towards the intervention. Before implementation, employees had a high commitment and rated all intervention components as appropriate, including high hopes for reduction of work load and inconvenient ways of communication. After implementation, commitment towards the intervention components directly connected to patient care (managed follow-up care, primary contact, case management) was still high, and the components were deemed appropriate. But a lack of adoption developed towards the use of the two software programs. The initial idea was still assessed positively, but the implementation and application were not considered helpful. Overall, high fidelity was anticipated, which could in fact be achieved, with the exception of procedures regarding the patient allocation platform and the data entry software.

### Domain associations

**Fig. 1:**
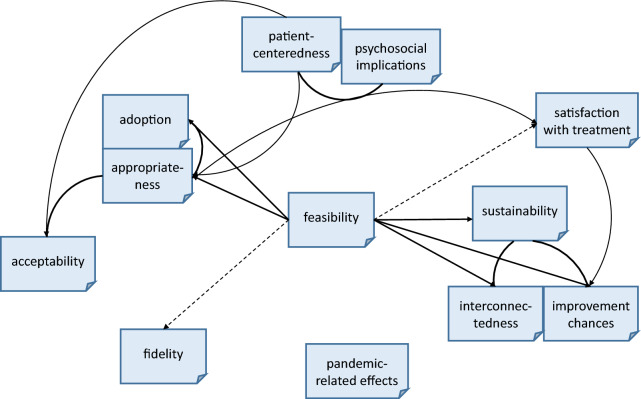
Domain interrelations. The solid lines represent strong connections and interdependencies between categories, while the dashed lines represent weaker connections between categories

The qualitative content analysis allowed to detect important connections and interdependencies between the analyzed domains. As seen in Fig. 1, feasibility, appropriateness, and patient-centeredness have been found to be central and decisive for several other domains.

In this study, appropriateness depended on feasibility. Further, strongly connected categories were patient-centeredness, psychosocial implications, and appropriateness. The latter exercised a mediating effect from the cluster of patient-centeredness and psychosocial implications to an increased adoption. These effects persisted even when an impaired feasibility and the need for adjusting fidelity became apparent. More detailed results can be found in the supplements [Additional File 3].

## Discussion

This study evaluated the implementation and intervention effects of a cross-sectoral and coordinated follow-up treatment for stroke patients.

We found interrelations between implementation outcomes and intervention effects, while some were more prominent in the implementation process than others (Fig. 1). Feasibility and appropriateness played the most central roles. Moreover, patient-centeredness and psychosocial implications, two of the data-driven inductive categories, had an unexpectedly high importance and strong influence on other aspects. Other categories, like pandemic-related effects, were less connected and considered less important for the implementation process.

Regarding the implementation outcomes, the implementation of the StroCare intervention was highly accepted and rated appropriate. Sustainability was affected, not by high costs and a high workload but by impaired feasibility due to technical issues. The newly implemented electronic solutions and systematic documentation did not support sustainability. Nevertheless, acceptance, adoption, and appropriateness of the intervention remained high. The good feasibility of all other intervention components except the allocation platform contributed to this effect. Also, some of the intervention effects contributed to the high acceptance, adoption, and appropriateness. Positive effects of the use of patient-reported outcome measures (PROMs), including the feeling of being cared for, could be found in the StroCare project. Similar results have been reported in a previous study, where the implementation of PROMs during acute care in the stroke unit was conducted and evaluated [28]. Positive effects on patient-reported health were found due to the PROMs themselves, the patients’ feeling of being cared for, and the additional attention of HCPs to patients. Further, in the previous study a need for simple procedures, systematic documentation within the electronic patient record, and better central electronic documentation was voiced. Even though the StroCare intervention was planned to integrate an improved electronic allocation and communication system between acute care and rehabilitation clinics, this specific intervention component did not prove to be sustainable.

A common barrier to implementation of stroke care interventions is that the innovations are not put into practice as planned, i.e., an impaired fidelity [29]. Additionally, high complexity and difficult adaptation of interventions to clinical practice hinder implementation and might cause problems with fidelity [29]. One of the main obstacles with the StroCare intervention was the use of the patient allocation platform and the use of the data entry software. Both programs were late to the start of implementation and had malfunctions at the beginning. At the same time, in line with the described common barriers in many stroke implementation programs, they were perceived as unpractical and incompatible with routine procedures. In this study, poor feasibility did not only impair fidelity but also adoption and appropriateness. However, the criticism regarding appropriateness was directed at the implementation and certain characteristics of the software and not at the idea of the software itself. In summary, this indicates that the idea of a platform for transmitting data and rehabilitation options is still considered to be very valuable, but the programs that were used in our study need to be revised.

In a review by Cormican and colleagues [30], organizational and professionals’ barriers were differentiated. Organizational barriers were lack of resources, of time, and of organizational processes, while professionals’ barriers were identified as limited knowledge and skills [30]. Neither of these commonly reported barriers occurred as decisive in the present study.

An important facilitator of satisfaction with treatment and appropriateness on both the employees’ and patients’ part were the intervention effects on patient-centeredness and further psychosocial aspects. Both intervention outcomes were facilitated mostly by cost coverage, including a timely transfer to inpatient rehabilitation, primary contact, outpatient care management, and case management. A review on rehabilitation interventions for prevention and treatment of depression discussed the beneficial effects of goal setting and achievement throughout follow-up care [31]. To set treatment goals and check the progress towards these goals regularly was part of the outpatient care management and may explain the positive effects on the patients’ enhanced mood and feeling of self-efficacy. Also, during outpatient care management, patients were provided with follow-up appointments every six months, which could be another facilitator towards increased patient-centeredness. It is also likely to have increased treatment adherence and to have opened more opportunities for receiving physical an occupational therapy. Involvement in care and treatment have been shown to support meeting rehabilitation needs of stroke patients [32]. Further common unmet needs in stroke patients, amongst others, are managing low mood and emotions in general, feeling of insecurity, aids/adaption, nursing and medical care, and receiving insufficient stroke-related information [9]. These different psychological, stroke- and information-related problems match the results of this study’s outcomes. They were met by implementing case management, primary contact, and outpatient care management in the present study. Patients reported an increased feeling of security and control as well as the possibility to gain information and voice fears and questions on several occasions.

Possibilities for improvement were discussed in this study and addressed both the intervention and its implementation. Next to a wider network of cooperating clinics and outpatient health care providers, many ideas for an improved follow-up care regarded the patient allocation platform or IT interventions in general. Most of the time, technical solutions for improving communication as well as transfer of data and information for HCPs and patients were envisioned. One example was a digital patient platform, where reports and patient information could be downloaded. Reviews reveal that barriers to e-health services are common and include difficulties in usability, fit of the design, and adaption to local software programs [33–35]. Developing a sustainable solution to current problems of e-health services seems to be a key element to an improved implementation.

A central limitation of this study is the inability of some patients to distinguish between the modified follow-up care after implementation of the StroCare intervention and usual care. However, stroke and related health care are unique experiences for most patients, so that an internal reference point of what usual care should actually be is probably missing. Fortunately, in controlled trials, this issue is unlikely to impair evaluation. This problem did not arise with the participating employees. Further, an interviewer bias cannot be ruled out, as the interviewers were part of the project team and responsible for the evaluation.

One of the study’s strengths is the equal stratification of patients and employees from the acute care and rehabilitation clinics. Moreover, we applied a before-after design for this study. Employees were interviewed before and one year after implementation (patients could not be interviewed before implementation). Another strength was the cross-sectoral approach of the project, which is reflected by the fact that patients and employees from eight different clinics and employees from four different professional groups could be included in the sample.

## Conclusions

The process evaluation of the StroCare intervention revealed positive and successful outcomes of implementation, whereas barriers were mostly due to problems concerning feasibility. Patient-centeredness and psychosocial implications were prominent and deemed very important, which indicates a high need for patient-centered and psychosocial interventions in the follow-up care of stroke patients.

## Supplementary Information

Additional file1.

Additional file2.

Additional file3.

## Data Availability

The datasets generated and analyzed during this study are not publicly available due to risk of identifiability of the participants and missing consent to publication.

## Abbreviations

HCPs: Health care professionals
IT: Information technology
SD: Standard deviation
PROMs: Patient-reported outcome measures
