# Supplementary material for: Process evaluation of a cross-sectoral, coordinated follow-up care of stroke patients: a qualitative study
Source: Neurol Res Pract. 2025 Jan 23;7:4. doi: 10.1186/s42466-024-00360-1 (PMC11755855; doi:10.1186/s42466-024-00360-1)
Supplement: Supplementary file 1 — Additional file1. [file 42466_2024_360_MOESM1_ESM.pdf]

Additional File 1. Semi-structured interview guides

Process evaluation of a cross-sectoral, coordinated follow-up care of stroke patients: a qualitative study

Theresa Schrage, Claudia Glissmann, Götz Thomalla, David Leander Rimmele, Holger Schmidt, Michael Rosenkranz, Stefan Boskamp, Martin Härter, Levente Kriston

Neurological Research and Practice

Introduction interview with employees before implementation

Thank you for agreeing to take part in this interview. [*Explanation, reference to audio recording, consent*]. We are conducting interviews with you and other employees of the cooperating clinics in the StroCare project, as well as with patients.

Among other things, the StroCare project aims to optimize four different aspects of follow-up healthcare for stroke patients:

- ensuring rapid allocation to inpatient rehabilitation clinic after acute care (cost coverage and electronic rehabilitation portal)
- the allocation of a case manager
- the allocation of a direct contact person for two years, Stroke Nurse, and
- ensured planning of outpatient care with regular follow-up appointments

Not only is the health care for stroke patients supposed to improve but also the patient's quality of life.
Our aim in these interviews is to evaluate the interventions of this stroke care. To this end, we are conducting interviews with several employees from various departments before and during the implementation of the interventions. This allows us to scientifically evaluate the implementation process and also gives us the opportunity to make any necessary adjustments to the StroCare implementation procedures.
In this interview we will talk about your opinion of the intervention components before implementation of the new stroke care.

Interview guide

Key question (narrative initiation)	Further questions	Specific questions	Questions for continuity and navigation
Part 1<br>Introducing questions<br>**Were you aware of the various components of the interventions and do you have any questions?**<br><br>**How is the access to health care to date for patients and what could change as a result of the StroCare intervention?**			
Part 2<br>Acceptability	Have there already been negative experiences with the implementation of	What do you think of the interventions components? Would you rate them rather positive or negative? Why?	Can you explain this in more detail?<br><br>*Paraphrase and summarize*

**How high do you rate the acceptance (among colleagues and employees) of the changes resulting from the intervention implementation?**<br><br>**In terms of the four different intervention components (immediate coverage of costs and rehab portal, case management, direct contact persons, outpatient aftercare), how useful do you think they are for improving patient care?**	interventions that could influence acceptance? What were they?	How will you and your colleagues receive the implementation of the new intervention and the changes to the routine in your department?
Part 3<br>Adoption<br>**Would you say that the people responsible for implementing the intervention (i.e. nursing staff, doctors, IT staff, ...) are committed to it?**<br><br>**To what extent do you think that commitment differs between the four intervention components?**	How strong is the motivation to implement the interventions? (Why so high, so low?)	Have there already been problems with the implementation of interventions or projects?<br><br>In your opinion, to what extent will the interventions be implemented?
Part 4<br>Appropriateness<br>**How helpful do you think the new intervention components are for the care of stroke patients?**<br><br>**Would you consider another intervention more appropriate?**	What is particularly important to you?<br><br>How helpful are the intervention components?	Will the additional (time) effort be appropriate?

Part 5<br>Feasibility<br>**Now I would like to talk to you about the feasibility of implementing the intervention in stroke care. Do you think that it will be possible to implement them in routine care in terms of structural and personnel conditions? What are the barriers and what are the facilitators?**<br><br>**In particular, do you think that the stroke nurse will have enough time to maintain contact with patients over the three-month period?**<br><br>**How do you rate the use of the patient allocation platform?**	What would support implementation and what would hinder it?<br><br>Do the clinics have sufficient resources to implement the interventions?	Do you feel sufficiently prepared for the introduction of the interventions?	
Part 6<br>Fidelity<br>**What do you think, will the direct cost coverage, the assistance from a case manager, the three-month contacts with the stroke nurse and the six-month follow-up appointments be implemented as planned?**	Will it be possible to adhere to the project's implementation guidelines?<br><br>What is your experience with the implementation of guidelines from previous projects?		
Part 7<br>Patient-centeredness<br>**What effects on patients and the Health Care Professionals do you expect?**<br><br>**Do you think that the intervention will have an impact on communication between Health Care Professional and patient? For example, will a patient be able to make a more informed decision?**	Will the patient-centeredness be strengthened?<br><br>Is there an impact on communication or the clinician-patient relationship?<br><br>Do patients and Health Care Professionals engage in shared decision-making?<br><br>Expected impact on quality of life?	What will change for patients, could you name an example?<br><br>How will the intervention be received by the patients?	

Part 8<br>Satisfaction with treatment<br>**What does "good health care" (of stroke patients) mean to you?**	In your opinion, can the care for stroke patients be improved with the interventions of the StroCare project?	Are there any aspects in the healthcare of stroke patients that you would like to change?<br><br>Are you satisfied with the changes in the healthcare situation?
Part 9<br>Pandemic-related effects<br>**How do you expect the COVID-19 pandemic to affect the intervention and its implementation?**	What differences could there be compared to the healthcare situation before the coronavirus pandemic?	
Part 10<br>Additional questions		Would you like to mention anything else that came to your mind during the interview?

Introduction interview with employees after implementation

Thank you for agreeing again to take part in this interview. [*Explanation, reference to audio recording, consent*]. We already talked before the implementation of StroCare what would be important during the implementation, what could be possible hindrances and facilitators.

In this phase of the project after intervention implementation, we are again conducting interviews with you and other employees, as well as stroke patients. [*If need be, short explanations of the intervention components*.]:

- ensuring rapid allocation to inpatient rehabilitation clinic after acute care (cost coverage and electronic rehabilitation portal)
- the allocation of a case manager
- the allocation of a direct contact person for two years, Stroke Nurse, and
- ensured planning of outpatient care with regular follow-up appointments

With these interviews, we want to evaluate the implementation and intervention of StroCare. The intervention components were initiated about a year ago and now the focus is on how this introduction worked, what helped and what hindered it.

Key question<br>(narrative invitation)	Further questions	Specific questions	Questions for continuity and navigation
Part 1<br>Introducing questions<br>**Do you have any questions about the interview or the interventions?**<br><br>**In your opinion, what has changed the most as a result of the newly introduced StroCare intervention?**			
Part 2<br>Acceptability<br>**How high is the level of acceptance (among colleagues) for the changes resulting from the new intervention?**	How high was the acceptance in the beginning of intervention and how is it now?	What is your assessment towards the interventions now? Rather positive or negative? Why?<br><br>How did you and your colleagues experience the introduction of the	Could you explain this in more detail?<br><br>*Paraphrasing and summarizing*

		StroCare intervention and changes of procedures?
Part 3<br>Adoption<br>**How committed are the people involved (i.e. nursing staff, physicians, IT staff, etc.), now that the interventions components have been implemented?**<br><br>**To what degree does the commitment differ between the four intervention components?**	How strong was the motivation/engagement to implement the intervention? (Why so high, so low?)<br><br>Was there or is there a difference in commitment between the four intervention components?	Did previous negative or positive experiences impact the intervention implementation?
Part 4<br>Appropriateness<br>**How helpful do you think the new intervention is for follow-up care of stroke patients?**	What is particularly important to you?<br><br>How helpful are the intervention components?<br><br>Are there other interventions that would be helpful or would also be suitable?	Could the benefits outweigh the costs?
Part 5<br>Feasibility<br>**Now, I would like to talk to you about the feasibility of the implementation. How did the realization into routine care develop in terms of structural and personnel conditions? What were and are barriers and what are facilitator?**<br><br>**In particular, do you think that the stroke nurse has enough time to maintain the three-month contact with the patients?**<br><br>**How is the handling of the rehab portal?**	What helped and what hindered implementation?<br><br>Do the clinics have sufficient resources to implement the intervention?	What else would have been for realization of the implementation?<br><br>In your experience, to what extent have the intervention components been implemented?
Part 6<br>Fidelity	Were changes to the guidelines necessary?	What are your experiences, were the immediate cost coverage, the assistance

**Is it possible to adhere to the project's implementation guidelines?**		from case management, the three-month contacts with the stroke nurse and the six-month follow-up appointments implemented as planned?
Part 7<br>Sustainability<br>**What is your opinion on how well the four different intervention components can be sustained in the future?**	What would improve sustainability?	Have you been able to gain experience in the past year that points to the potential for sustainability?
Part 8<br>Patient centeredness<br>**What impact have you observed on patients and the Health Care Professionals?**<br><br>**Have you noticed whether the intervention has an impact on communication between the Health Care Professional and the patient?**	Is patient-centeredness being strengthened?<br><br>Is there an influence on communication or the relationship between Health Care Professional and patient?<br><br>Do patients and Health Care Professionals participate in shared decision-making?<br><br>Did you observe any effects on quality of life?	What has changed for patients?<br><br>How do the patients experience the intervention components?
Part 9<br>Satisfaction with treatment<br>**How satisfied are you with the current stroke care?**	To what extent has the treatment of stroke patients changed?	In your opinion, can the care for stroke patients be improved with the StroCare intervention?
Part 10<br>Pandemic-related effects<br>**What influence do the effects of the Covid19 pandemic have on intervention and their implementation?**	What differences are there compared to the health care situation before the pandemic?	
Part 11<br>Additional questions		Would you like to mention anything else that came to your mind during the interview?

Introduction interview with patients (after implementation)

Thank you for agreeing to take part in this interview. [*Explanation, reference to audio recording, consent*]. We are conducting interviews with you and other patients, as well as with clinic employees who are participating in the StroCare project. StroCare aims to improve the healthcare of stroke patients. To this end, four different innovations in care have been introduced, some of which you may already be familiar with:

- ensuring rapid allocation to inpatient rehabilitation clinic after acute care (cost coverage and electronic rehabilitation portal)
- the allocation of a case manager
- the allocation of a direct contact person for two years, Stroke Nurse, and
- ensured planning of outpatient care with regular follow-up appointments

Not only is the health care for stroke patients supposed to improve but also the patient's quality of life. Our aim in these interviews is to assess the innovations that have already been introduced to stroke care. We want to know what has worked out and what did not.

Key question<br>(narrative invitation)	Further questions	Specific questions	Questions for continuity and navigation
Part 1<br>Introducing questions<br>**Have you ever participated in an interview?**<br><br>**Do you have questions about the interview or the innovations discussed?**			
Part 2<br>Appropriateness<br>**How helpful and appropriate do you think the innovations are for the health care of stroke patients?**	What is particularly important to you?<br><br>How helpful are the innovations?<br><br>Are there any other aspects that you would also find helpful and appropriate?	How appropriate do you find the follow-up care by the stroke nurse and the outpatient follow-up care at the acute clinic?<br><br>How helpful did you find the case management?	Can you explain this in more detail?<br><br>*Paraphrasing and summarizing*
Part 3<br>Feasibility<br>**Having just talked about the benefits of the new innovations, there may also be additional effort involved. Is this effort manageable?**	Hindering and facilitating factors?	Did you get along with the additional effort due to the new follow-up care program? (contacts with the stroke nurse, case management, follow-up appointments)	

Part 4<br>Patient-centeredness<br>**How accessible would you say the outpatient follow-up care is for you?**<br><br>**To what degree were your needs addressed through contact with the stroke nurse and case management? Were they helpful?**<br><br>**Have you noticed whether the innovations have had an impact on the communication between you and the practitioners?**	Is the patient being prioritized more in the course of the treatment?<br><br>Observed effects on quality of life?<br><br>Is there an impact on communication or the relationship between a patient and a Health Care Professional?	What has changed for patients?<br><br>Did you feel well taken care of during follow-up care?<br><br>To what extent were your needs addressed through contacts with the Stroke Nurse and Case Management? Were they helpful to you?<br><br>Were you involved in decisions to the extent you desired/could you participate to the extent you wished?
Part 5<br>Satisfaction with treatment<br>**What does good health care (for stroke patients) mean to you?**	How satisfied are you with the current health care?	
Part 6<br>Pandemic-related effects<br>**Now we will talk about something that has been affecting all of us for a while now. With regard to the impact of the coronavirus pandemic, what influence has this had on the StroCare innovations and therefore the care of stroke patients?**	What differences are there compared to the healthcare situation before the pandemic?	
Part 7<br>Additional questions		Would you like to mention anything else that came to your mind during the interview?
