# Supplementary material for: Process evaluation of a cross-sectoral, coordinated follow-up care of stroke patients: a qualitative study
Source: Neurol Res Pract. 2025 Jan 23;7:4. doi: 10.1186/s42466-024-00360-1 (PMC11755855; doi:10.1186/s42466-024-00360-1)
Supplement: Supplementary file 2 — Additional file2. [file 42466_2024_360_MOESM2_ESM.pdf]

Additional File 2. Detailed methods

Process evaluation of a cross-sectoral, coordinated follow-up care of stroke patients: a qualitative study

Theresa Schrage, Claudia Glissmann, Götz Thomalla, David Leander Rimmele, Holger Schmidt, Michael Rosenkranz, Stefan Boskamp, Martin Härter, Levente Kriston

Neurological Research and Practice

Measurements

Sociodemographic data were gathered by self-report. Patients provided information about their gender, age, living situation, level of education, employment situation, partnership, marital status, and number of children. Employees were asked about their age, profession, task during this study, and work experience in years.

A semi-structured interview guide was developed according to Helferrich [1] (see Supplement A). Questions were based on the proposed indicators for implementation analysis by Proctor and colleagues [2]. Applied indicators were acceptance, adoption, appropriateness, feasibility, fidelity and sustainability. Furthermore, questions regarding the patient-centeredness of the interventions, as well as satisfaction with the treatment and effects on the implementation due to the Corona-pandemic were included. Patient-centeredness was understood as in the integrative model described by Scholl and colleagues [3], which has 15 interrelated dimension. Most of these dimensions address the encounter between a HCP and a patient and involve, among others, the clinician-patient relationship, a patient’s access to care, and a biopsychosocial perspective.

Analysis

Digitally recorded interviews were transcribed using the software F4 [4]. Only verbal information was included in the transcripts. The transcripts of the interviews were structured using MAXQDA (version 24), a software for organization and visualization of qualitative data [5]. The analysis was conducted using qualitative content analysis based on Mayring [6]. First, the category system was developed (categories derived from literature). TS and CG both coded the same transcript, reviewed the resulting categories and agreed upon a category system (categories derived from literature and from the transcripts). Subsequently, all transcripts of the patient and employee interviews were coded using the same category system. To test intercoder reliability, TS and CG independently coded the same six transcripts per group of interviews. An adequate intercoder reliability war reached with a rate of matching codings of 70% [7].

The deductive-inductive approach was applied to define categories. Deductive categories were chosen beforehand based on the implementation analysis framework from Proctor and colleagues [2], acceptance, adoption, appropriateness, feasibility, fidelity and sustainability. In addition, categories of intervention effects were included beforehand: patient-centeredness, satisfaction with treatment, and pandemic-related effects. Inductive categories were derived from the text material during qualitative content analysis. This approach allows to enhance the theoretical framework. During the initial coding of the first text material, three additional inductive categories emerged: psychosocial implications, interconnectedness, and potential for improvement. The category system is presented in Table 1.

References

1. Helfferich, C. (2009). *Die Qualität Qualitativer Daten*. Wiesbaden: VS Verlag für Sozialwissenschaften.
2. Proctor, E., Silmere, H., Raghavan, R., Hovmand, P., Aarons, G., Bunger, A., … Hensley, M. (2011). Outcomes for Implementation Research: Conceptual Distinctions, Measurement Challenges, and Research Agenda. *Administration and Policy in Mental Health*, *38*(2), 65–76. https://doi.org/10.1007/s10488-010-0319-7
3. Scholl, I., Zill, J. M., Härter, M., & Dirmaier, J. (2014). An integrative model of patient-centeredness - a systematic review and concept analysis. *PloS One*, *9*(9), e107828. https://doi.org/10.1371/journal.pone.0107828
4. Audiotranskription - f4transkript. (2023). Retrieved from https://www.audiotranskription.de/f4transkript/
5. MAXQDA. (2023). Retrieved June 1, 2023, from https://www.maxqda.com/de/
6. Mayring, P. (2008). *Qualitative Inhaltsanalyse: Grundlagen und Techniken*. Weinheim: Beltz.
7. O'Connor, C., & Joffe, H. (2020). Intercoder Reliability in Qualitative Research: Debates and Practical Guidelines. *International Journal of Qualitative Methods*, *19*, 1609406919899220. https://doi.org/10.1177/1609406919899220
