# Supplementary material for: Process evaluation of a cross-sectoral, coordinated follow-up care of stroke patients: a qualitative study
Source: Neurol Res Pract. 2025 Jan 23;7:4. doi: 10.1186/s42466-024-00360-1 (PMC11755855; doi:10.1186/s42466-024-00360-1)
Supplement: Supplementary file 3 — Additional file3. [file 42466_2024_360_MOESM3_ESM.pdf]

Additional File 3. Detailed results

Process evaluation of a cross-sectoral, coordinated follow-up care of stroke patients: a qualitative study

Theresa Schrage, Claudia Glissmann, Götz Thomalla, David Leander Rimmele, Holger Schmidt, Michael Rosenkranz, Stefan Boskamp, Martin Härter, Levente Kriston

Neurological Research and Practice

Table. Representative quotes from patients and staff members

Category	Quote
Acceptability	"The portal can still be improved. I think the details can still be polished a bit. But overall I think it was good." (*B3-2staff*)
Adoption	"Very well, in fact. So the doctor, you can really tell that this is a doctor for whom the patients, that is, for whom people are important." (*B7patient*)
Appropriateness	"When the patients are at home, not everything is clear to them about their disease and how they should deal with it. Some are better connected through relatives and therapists and others not. So that when the patients come to the check-ups after six months, one has very different starting points with each and can then again provide a lot of education to bring all patients up to speed as good as possible. What is important in stroke follow-up care and what they are already doing correctly, what they can still do, that is inquired about here [StroCare outpatient care management] clearly specified and in a completely standardized way." (*C4-2staff*)<br><br>"That is, this interaction from a professional, diagnostic and medical point of view, to see one's own personal living conditions very well in the context of the health condition and still to find a balance of interests. This is what has been achieved." (*PA6patient*)
Feasibility	"It arrived far too late, never really got going and by the time it was running reasonably well, everyone had lost their drive." (*C1-2staff*)
Patient-centeredness	"I was asked and I had an impact, I said I had problems and then it was taken care of right away." (*PC4patient*)<br><br>"Patients have more of a feeling that they are part of the treatment, of the success of the treatment. It's not so externally determined, but rather when their subjective assessment influences the outcome, so to speak, and thus also influences the course of the therapy, then I think they feel more on board with the treatment concept." (*A4-2staff*)
Satisfaction with treatment	"I was thrilled, if I may say so." (*PA4patient*)
Psychosocial implications	"That ensures security when you can have these regular examinations and conversations." (*PA1patient*)

Acceptability

Acceptance of the StroCare interventions was generally neutral to positive. Patients often could not distinguish the modified care (StroCare intervention) from usual care. Although patients with a history of a previous stroke viewed the interventions appropriate and beneficial. The outpatient care management in particular achieved a high level of acceptance among patients. HCPs and IT staff involved in StroCare in general indicated a high level of acceptance of the intervention. Nevertheless, the software used for the electronic allocation of patients and for data entry of the patient-reported outcome measures (PROM), respectively, were seen somewhat critically. Technical problems, delays in implementation, and low feasibility of the software programs were mentioned as the primary sources of concern. A positive influence on acceptance was brought by a sufficient flow of information and planning of resources within the treatment teams of each clinic, and by the perceived positive effects on patient interaction and the treatment of patients.

> "The portal can still be improved. I think the details can still be polished a bit. But overall I think it was good." (*B3-2staff*)

Adoption

There was a general commitment to the implementation of the StroCare intervention, which was often explained by the perceived usefulness of the intervention components for patient care.

> "Yes, so we all work for the good of the patient and if we have the opportunity to actively help them [with these intervention components], we do that, yes of course." (*B5-1staff*)

> "Very well, in fact. So the doctor, you can really tell that this is a doctor for whom the patients, that is, for whom people are important." (*B7patient*)

Nevertheless, the high effort required to use the patient allocation platform and the data entry software, as well as the necessity of a selective inclusion of patients compromised the generally high adoption by employees. Since only patients of one health insurance company could be enrolled in the study, in part it was difficult to develop a continuous process. This in turn had an effect on adoption.

> "That will then be forgotten, that won't happen, no one will check [the allocation platform]." (*C1-2staff*)

> "I don't think we've seen the benefits yet; the costs still outweigh the benefits. But I think it is reasonably predictable that the benefit will be higher than the effort, that we will have a benefit." (*A4-2staff*)

Appropriateness

One of the most important categories among the implementation outcomes was appropriateness. Several components of the StroCare intervention were deemed highly relevant and compatible to the patient's situation and needs after the stroke incident. These were the continuous support and guidance throughout the aftermath of the incident, specifically referring to the outpatient follow-up care at the acute care hospitals and the case management.

“When the patients are at home, not everything is clear to them about their disease and how they should deal with it. Some are better connected through relatives and therapists and others not. So that when the patients come to the check-ups after six months, one has very different starting points with each and can then again provide a lot of education to bring all patients up to speed as good as possible. What is important in stroke follow-up care and what they are already doing correctly, what they can still do, that is inquired about here [StroCare outpatient care management] clearly specified and in a completely standardized way.” (*C4-2staff*)

A seamless transition both from acute care to inpatient rehabilitation and from inpatient rehabilitation to outpatient care was also positively highlighted by patients. Also, the outpatient appointments every six months were perceived as relevant and supportive in regard to the symptom burden and arising questions. Not only did they provide predictability and orientation in a situation of great change, but also a regularly provided space for questions and addressing fears. This in turn led to a better understanding of the disease itself and any necessary changes to lifestyle and medication.

“That is, this interaction from a professional, diagnostic and medical point of view, to see one's own personal living conditions very well in the context of the health condition and still to find a balance of interests. This is what has been achieved.” (*PA6patient*)

“That is a huge help. I have the feeling that I'm in good hands and that I'm being positively guided somewhere.” (*PC4patient*)

Furthermore, patients perceived the medical examinations during the StroCare intervention to be appropriate and different to treatment in a private practice.

“It's very good, because the examination there [acute care hospital] is, how shall I say it, much more extensive than when I go to the doctor here [private practice], to the neurologist and to the general practitioner. […] I think the most important thing for me personally is that the aftercare is provided. That was not the case in the past when someone had a stroke.” (*QPB2patient*)

On the other side, the interventions also were sometimes deemed unnecessary in cases of a mild stroke.

“I can't say whether that was helpful or whether it was just a normal conversation.” (*PC5patient*)

Additionally, for the staff, implementation was not appropriate in every regard. The patient allocation platform was initially deemed appropriate but in the end it has been found to be rather hindering and not supportive of information flow.

Feasibility

The time required for and the pacing of the appointments were considered manageable by patients. Also, because the outpatient appointments were spread across three different hospitals, the distances to the acute care hospitals for the follow-up appointments were considered short or familiar.

“No effort at all.” (*PB1patient*)

On the contrary, hospital staff assessed the feasibility of the intervention as limited. Two main barriers were the two software programs for patient allocation and for data entry, as well as the selective inclusion of patients into the study.

> "At the moment, it's actually more of an extra effort, because there are only relatively few cases and you have to maintain an extra internal structure for them." (*G2-2staff*)

The selective inclusion of patients and the study environment were problematic for the routine procedures in the clinics because the enrollment and timely transfer to the rehabilitation clinics were more difficult. Some patients could not be asked for informed consent up to three days after admission, as acute stroke treatment can require extensive testing. In usual care, the case management would already have initiated the first steps for follow-up care and inpatient rehabilitation. Furthermore, selective inclusion in the study was a barrier to developing routine procedures. Because of the small number of patients who were eligible for the StroCare intervention in relation to all stroke patients, procedures were not carried out regularly and by every employee. This led sometimes to halting communication.

The second barrier to a feasible implementation were the software programs.

> "It has been a disaster." (*C1-2staff*)

> "It arrived far too late, never really got going and by the time it was running reasonably well, everyone had lost their drive." (*C1-2staff*)

Problems with the software programs included a delayed implementation, inconvenient registration process, counterintuitive interface, and technical difficulties. Because of the delayed implementation and system errors, PROMs could not be entered directly into the according software. The outcomes had to be recorded on paper and entered into the software later, adding to the employees' workload.

> "And we have also had some technical problems. So when there was a patient, the program didn't work, or we tried to register a patient and then he was rejected by the rehabilitation clinics and we didn't get a response quickly enough, so the patient had to be referred to a rehabilitation clinic in the normal way." (*C4-2staff*)

> "This means that you are basically stuck in the same waiting game that you would normally be in with a conventional procedure." (*E6-2staff*)

Similar to the communication between the acute care and rehabilitation clinics, the information flow with the case management was affected. Due to the inconvenient usability of the rehabilitation platform, the reports were sometimes entered late or only occasionally.

Despite the problems regarding the software programs and selective inclusion of patients, the other StroCare intervention components could be successfully carried out. The contact to the patients every three months made by a study nurse, the follow-up appointments, and the contacts between the case manager and the patients were not affected by the technical challenges. Feasibility regarding these elements was reported to be high.

Fidelity

For the most part, the intervention components were carried out according to protocol. Minor changes had to be made to the automatic cost coverage of the health insurance companies for inpatient rehabilitation. Cost coverage for patients with a low functional impairment now required an application for inpatient rehabilitation.

> "Minimal changes, but yes that is entirely acceptable. It's not a big problem." (*A4-2staff*)

Changes with a greater impact were made regarding the two software programs. In case of malfunctions using the electronic patient allocation platform, HCPs from both the acute care and rehabilitation clinics used the phone or e-mail to make contact. The PROMs had to be documented using pen-and-paper since the software for data entry was delayed and showed malfunctions even after implementation.

Sustainability

All StroCare intervention components were deemed sustainable with the exception of the patient allocation platform. Results on the rehabilitation management were ambivalent, and the allocation platform was suggested to be omitted or substantially revised. According to the participants, important facilitators of a successful continuation would be the integration of the intervention into routine care, including an extension to all stroke patients and the provision of additional resources in form of personnel.

Patient-centeredness

> "I was asked and I had an impact, I said I had problems and then it was taken care of right away." (*PC4patient*)

> "Patients are often shocked by their own disease, especially when they are severely affected […], there is something happening in the body, which also means to the soul. I believe that patients in this situation feel safe and in good hands in the presence of people who care about them." (*C2-1staff*)

Patient-centeredness was a major principle in the StroCare project. The StroCare intervention was reported to have positively influenced several dimensions of patient-centeredness, such as the clinician-patient-relationship, seeing the patient as a unique person, integration of medical and non-medical care, access to care, patient involvement in care, and emotional support. The changes offered the possibility to develop trust between the patient and the HCPs and provided a primary contact for each patient, not just to one person but also to a whole institution. This long-term primary contact point went hand in hand with the perception of a shared responsibility for the patient's health. Both the patients' and the HCPs' perspective changed during the StroCare project. Patients were involved in the treatment to a higher degree and considered themselves to be able to influence treatment success more strongly. HCPs were moving forward from a necessary focus on medical data, which is frequently called for in acute care, to a more holistic view of care.

> “Patients have more of a feeling that they are part of the treatment, of the success of the treatment. It's not so externally determined, but rather when their subjective assessment influences the outcome, so to speak, and thus also influences the course of the therapy, then I think they feel more on board with the treatment concept.” (*A4-2staff*)

> “And for the practitioners, it also changes the assessment a certain amount that you don't just look at key numbers in the lab or whatnot, but that you also have the awareness that it's also about the person managing their life.” (*A4-2staff*)

The relationship to the study nurses, neurologists, and case manager was described as positive. According to patients, HCPs were attentive, interested, versed in the patient’s case, taking patients seriously, engaged, focused on the patient and the conversation with them, taking time, asking and answering questions, and involving patients in medical decisions.

> “And this situation, this decision could not be taken from me by the physician, but the medical environment could provide me with the means, i.e., data and expertise, which put me in a position to weigh up these goods for the better of me and for my health.” (*PA6patient*)

The use of PROMs during outpatient care management reinforced the impression among patients that they are taken seriously, that the HCPs are thorough, and that they are interested in the patient’s case and the patient’s view of symptoms.

> “Not only a number.” (*PB2patient*)

Satisfaction with treatment

The patient’s satisfaction with treatment overlapped with the category of appropriateness, patient-centeredness and psychosocial implications.

> “All other things no longer run by cardiologists, but rather by (one of the acute care hospitals), because there I am in a place that also has, I say, this in one place and therefore, I say, I am also quite well cared for.” (*PA7patient*)

> “I was thrilled, if I may say so.” (*PA4patient*)

For members of the staff, satisfaction with treatment was equivocal. In part, the modified follow-up care was met with an overall satisfaction, especially concerning the improved neurological care after rehabilitation. At the same time, standard stroke care in Northern Germany was viewed as already having a high quality. Also, dissatisfaction due to the omnipresent lack of personnel in the health care system in general influenced the assessment of treatment satisfaction.

Psychosocial implications

Several psychosocial effects seem to be connected to the StroCare intervention, which were emphasized especially by the patients. These effects were the reduction of fear and of the feeling of loneliness, an increase in the sense of security and of a competence to act, mood enhancement and a stronger feeling of control, as well as a reduction of stress and of the feeling of vulnerability.

> “That ensures security when you can have these regular examinations and conversations.” (*PA1patient*)

Patients and staff members explained these effects by various mechanisms. The pre-planned and set structure of the follow-up care, as well as the medical examinations enhanced patients’ feeling of control and decreased the perceived health-related stress. Having a primary contact person and consequently the possibility of building a trustful relationship enabled the patients to feel secure in their treatment.

> “It is one of the most important points for me. You're very insecure and vulnerable after [stroke] and you need people. Of course, your social life also helps but especially in this clinical environment, you need people who give you security. And if it's always the same [health care professional], and that worked great for me, then I felt very safe.” (*PC6patient*)

> “Compliance increases significantly and the treatment improves as a result, also in terms of these hard facts, that the risk factors are better controlled, so there are fewer recurrences and this alone improves the quality of life, that you don't have a new stroke.” (*A4-2staff*)

One mechanism that was further emphasized by the patients were the frequent occasions to ask questions and to address their fears. Essential to that point is that the participants did not have to initiate contact to address their questions and fears. Mostly, in usual care, these questions and fears would not have been brought to attention of the HCPs because patients perceived them as minor or as cumbersome. But these occasions to meet and converse enhanced the patients’ feeling of security and reduced feelings of fear, loneliness, vulnerability, and stress. Further, patients perceived the HCPs as dependable and appreciative, which had a positive influence on the patients’ mood and fear.

> “Attitude and appreciation and also communication. I'm going to be brutal about that. That brings about good humor and healing.” (*PC7patient*)

Interconnectedness

Between the three different parties involved in the follow-up care, the acute care hospital, the rehabilitation clinic, and the case manager, a higher interconnectedness and flow of information was expected. For the most part, this could not be achieved by the use of the patient allocation platform, which was supposed to be the key element for reaching this goal. Thereby, interconnectedness and information flow were not improved as much as anticipated. Also, staff members from the rehabilitation clinics noticed the lack of communication and exchange with the case management in general. However, a better exchange between acute care and rehabilitation clinics could be achieved by regular project meetings.

Pandemic-related effects

Regarding possible effects of the Corona pandemic, few changes in the care provided were noted but none had a major impact on the implementation of the intervention. During inpatient rehabilitation, a less amount of therapy could be offered than usual because smaller groups were necessary, and some follow-up appointments had to be cancelled due to Corona infection. A greater effort for patients was

required in that they had to wear masks and organize testing before follow-up appointments. However, patients found these efforts to be manageable.

Potential for improvement

Development opportunities were largely identified for IT-related topics of patient care. The patient allocation platform would be helpful as an application for tablets and including a direct link to the hospital information system. Then, all necessary patient information could be transferred directly from acute care to the rehabilitation clinic.

> "If this portal would also be accessible via an app, then it would be even easier in the end because the social worker could even go to the patient's bedside and while they are there check if a rehab is available somewhere." (*B9-1staff*)

Additionally, it was stated that other HCPs responsible for outpatient follow-up care, like physiotherapists, logotherapists, should have gained access to the patient allocation platform. Also, the inclusion of a chat feature on the platform would allow a better communication between the different HCPs without adding length to the original request for inpatient rehabilitation.

> "For example, the diagnosis of obesity. That's not a problem for an acute clinic, but it is for a rehabilitation facility, because if we have severely obese patients, we need two therapists for the therapy, as this is a completely different setting for us than if we have someone who is slightly overweight." (*E6-2staff*)

It was also suggested that the platform could be improved by integrating digital health applications for patients, for example the self-documentation of a patient's vital signs in form of a medical diary. In this scenario, HCPs at the acute care clinics could use this longitudinal assessment of the patients' health status. Furthermore, patient information was identified as another point of possible improvement. Suggestions included a digital platform for patients where they could look at or download patient information and treatment reports or look at stroke information videos. Also, a feature for exchange between stroke patients was suggested, for example, in form of a questions and answers forum.

Moreover, for a coordinated and long-term follow-up stroke care, the establishment of stroke outpatient centers was proposed. There, health care could be coordinated and/or provided after acute care and inpatient rehabilitation. At last, the expansion of the StroCare intervention to include more rehabilitation clinics in Northern Germany and to consider other health conditions than stroke was suggested.

Appraisal of intervention components

A summarizing overview of the appraisal of each intervention component derived from the content analysis is displayed in Table 4.

Table. Appraisal of intervention components

Categories	Primary contact	Outpatient care management	Case management	Coverage of costs	Electronic allocation of capacities
Acceptability	+	++	+	++	+
Adoption	+	++	+	+/-	-
Appropriateness	+	++	++	+	-
Feasibility	+	+	+	+	--
Fidelity	+	+	+	++	-
Sustainability	+	+	+	+	-
Patient-centeredness	+	++	++	+	+/-
Satisfaction with treatment	+/-	+	+	+/-	-
Pandemic-related effects	+/-	+/-	+/-	+/-	+/-
Psychosocial Implications	+	++	+	+/-	+/-
Interconnectedness	+/-	+/-	+	+/-	-
Potential for improvement*	+/-	+	+	+	++

Note: *The positive and negative signs in the table represent the overall assessment per category of an intervention component where: ++ = positive, + = mainly positive, +/- no or neutral assessment, - mainly negative, -- negative.*
** The positive and negative signs for this category are to be interpreted as that positive signs represent higher potential for improvement and negative signs represent no potential for improvement.*

Contrasting assessments before and after implementation

The views and expectations of the employees before implementation of intervention and one year after implementation differed. The categories adoption, appropriateness, feasibility, fidelity, and psychosocial implications indicate a change in attitudes towards the intervention. Before implementation, employees had a high commitment and rated all intervention components as appropriate, including high hopes for reduction of work load and inconvenient ways of communication. These expectations do not seem to have been fully met by implementing the intervention. The commitment towards the intervention components directly connected to patient care (managed follow-up care, primary contact, case management) was still high and also deemed appropriate. But a lack of adoption developed towards the use of the two software programs, of which the initial idea was still assessed positively, but the implementation and application were not considered helpful. Several aspects decreasing the intervention's feasibility were foreseen beforehand, including that routine procedures would take time to develop and that the inclusion of only one health insurance company would complicate procedures. Also, problems with the implementation of the patient allocation platform were anticipated, although

not to the degree in which the problems presented themselves afterwards. The awaited interruptions of the implementation due to high workload in acute stroke care did not arise. Overall, high fidelity was anticipated, which could in fact be achieved, with the exception to procedures regarding the patient allocation platform and the data entry software.

The assessment of psychosocial implications before and after implementation differed only slightly. The employees expected the intervention to contribute to a faster mobilization of patients and to a prevention of depressive symptoms during follow-up care. These issues did not arise in the interviews after implementation. However, implications were reported in the post-interviews that were not mentioned beforehand. These included the increase of the patients' feeling of control and the positive effect on self-determination by the specification of treatment goals with the patients in advance and that they were evaluated regularly during outpatient appointments. Intervention components were not seen to lead to a better communication and flow of information between the clinics as well as between clinics and the case manager as anticipated. However, HCPs did get more information about a patient's case after discharge through the allocation platform, even though, as stated before, the use of the platform was considered inconvenient. Most effects due to the Corona pandemic on the implementation were anticipated beforehand. The HCPs expected problems with the patient allocation to rehabilitation clinics, but these were not confirmed after implementation.

A regarding the other categories (acceptability, sustainability, patient-centeredness, satisfaction with treatment, improvement chances) did not show notable changes from before to after implementation.
